# Safe Autonomous Driving with Latent Dynamics and State-Wise Constraints

**DOI:** 10.3390/s24103139

**Published:** 2024-05-15

**Authors:** Changquan Wang, Yun Wang

**Affiliations:** 1 Institute of Microelectronics of the Chinese Academy of Sciences, Beijing 100029, China; wangchangquan@ime.ac.cn; 2 University of Chinese Academy of Sciences, Beijing 101408, China

**Keywords:** autonomous driving, safe reinforcement learning, latent dynamics, state-wise constraints, barrier function

## Abstract

Autonomous driving has the potential to revolutionize transportation, but developing safe and reliable systems remains a significant challenge. Reinforcement learning (RL) has emerged as a promising approach for learning optimal control policies in complex driving environments. However, existing RL-based methods often suffer from low sample efficiency and lack explicit safety constraints, leading to unsafe behaviors. In this paper, we propose a novel framework for safe reinforcement learning in autonomous driving that addresses these limitations. Our approach incorporates a latent dynamic model that learns the underlying dynamics of the environment from bird’s-eye view images, enabling efficient learning and reducing the risk of safety violations by generating synthetic data. Furthermore, we introduce state-wise safety constraints through a barrier function, ensuring safety at each state by encoding constraints directly into the learning process. Experimental results in the CARLA simulator demonstrate that our framework significantly outperforms baseline methods in terms of both driving performance and safety. Our work advances the development of safe and efficient autonomous driving systems by leveraging the power of reinforcement learning with explicit safety considerations.

## 1. Introduction

The advent of autonomous driving has sparked a technological revolution, promising a future where vehicles navigate complex environments with minimal human intervention. This transformation hinges on the ability of autonomous agents to perceive their surroundings accurately, make informed decisions, and execute precise control maneuvers. Despite significant progress, the development of reliable and safe autonomous driving systems remains a formidable challenge, particularly in dealing with the intricacies of real-world traffic scenarios.

Reinforcement learning has emerged as a pivotal approach in training agents to solve a variety of control tasks. Algorithms such as Deep Q-Networks (DQNs) [1], Proximal Policy Optimization (PPO) [2], and Soft Actor–Critic (SAC) [3] have been instrumental in enabling agents to learn from experience and improve their decision making over time. In the context of autonomous driving, reinforcement learning has been applied to learn optimal control policies through interaction with the environment. For instance, Ref. [4] used DQN to learn steering control for an autonomous vehicle in a simulated environment. Ref. [5] developed a continuous control deep RL algorithm to learn a deep neural policy for driving a vehicle on a simulated racing track. Ref. [6] developed a hierarchical deep RL framework to handle driving scenarios with intricate decision-making processes, such as navigating traffic lights.

However, traditional reinforcement learning algorithms face two main challenges: (1) They often assume a low-dimensional and structured state space. Yet, in real-world applications such as robotic vision or autonomous driving, state observations are frequently high-dimensional (e.g., images or videos), complicating the learning of effective strategies. (2) In safety-critical applications, considering safety merely at the trajectory level is insufficient. Traditional methods may fail to ensure safety at each state. We will elaborate on the current research tensions from these two perspectives and the work we have conducted.

Regarding high-dimensional observations, the high-dimensional and noisy nature of sensor data in autonomous driving poses a significant challenge for traditional RL algorithms. The “curse of dimensionality” often leads to inefficient learning and suboptimal policies. To tackle this, the concept of latent state space has been introduced, offering a solution by compressing high-dimensional sensory inputs into a lower-dimensional representation. Ref. [7] introduces Deep Planning Network (PlaNet), which is a model-based agent that effectively learns latent environment dynamics from pixel observations and accomplishes online planning to navigate complex control tasks with partial observability and sparse rewards. The Dreamer [8,9,10] series of algorithms represents a progression of model-based reinforcement learning approaches designed to improve the efficiency and effectiveness of planning in complex, high-dimensional environments. This approach has been successfully applied in RL for autonomous driving, enhancing sample efficiency and improving generalization capabilities. For instance, Ref. [11] introduces a model-free deep reinforcement learning framework tailored for urban autonomous driving scenarios, employing specialized input representations and visual encoding to capture low-dimensional latent states, which is demonstrated to be effective through complex roundabout navigation. Ref. [12] presents an interpretable autonomous driving framework utilizing latent deep reinforcement learning, which enables the generation of a semantic bird-eye view mask for explaining the decision-making process in complex urban scenarios. Ref. [13] maps high-dimensional images into implicit affordances using a pre-trained Resnet-18 encoder in a supervised setting.

Safety remains a paramount concern in autonomous driving. Traditional RL algorithms often lack inherent safety guarantees, which is unacceptable for safety-critical applications. To this end, State-Wise Safe Reinforcement Learning (SRL) [14] has been proposed, aiming to ensure that learned policies adhere to safety constraints at every state. These methods integrate safety directly into the learning process, preventing the agent from taking actions that could lead to unsafe states, which is vital for applications where safety violations have severe consequences. Ref. [15] optimizes the merging process of automated vehicles at traffic intersections, ensuring state-wise safety and efficiency by utilizing control barrier functions. Ref. [16] presents a novel framework that integrates differentiable control barrier functions into a neural network architecture, enabling end-to-end training and guaranteed safety in various driving tasks. Additionally, there are similar studies applying these concepts in the control of autonomous driving.

Targeting the aforementioned challenges, there has been research [17,18] that combines latent state space with state-wise safety to create a reinforcement learning framework capable of handling high-dimensional observational data while ensuring safety at each state. Their methods have improved the efficiency, safety, and generalization capabilities of the algorithms, making them more suitable for complex and safety-critical application scenarios. However, these studies have been implemented within the simulated games of Safe Gym [19]. To date, there has been no research in the field of autonomous driving that combines latent state space with state-wise safety. Therefore, in our study, we introduce a novel framework that bridges this gap by developing a latent space model and a barrier function that encodes state-wise PPO safety constraints. Our approach combines the benefits of model-based learning with the safety guarantees required for autonomous driving, resulting in a control policy that is not only efficient but also compliant with safety regulations. In contrast to existing works such as Roach PPO [20], which utilize custom BEV images as input for PPO-based agents, and Chen et al.’s works [11,12], which introduce model-free base latent state space and interpretable frameworks for autonomous driving without state-wise safety guarantees, our framework addresses the limitations of existing methods by providing a more comprehensive and proactive approach to safety without compromising the learning efficiency or the performance of the autonomous agent.

Our contributions encompass several aspects. Firstly, we propose a novel framework that effectively integrates latent state space modeling with state-wise safety constraints, addressing the limitations of high-dimensional sensory inputs and ensuring safety at each state. Secondly, our approach enhances the efficiency and generalization capabilities of autonomous driving systems, outperforming existing methods in both driving performance and safety. Thirdly, we conduct extensive experiments in the CARLA simulator, demonstrating the robustness and applicability of our framework in diverse driving scenarios. Lastly, our work contributes to the advancement of safe and reliable autonomous driving systems, paving the way for a future with minimal human intervention and enhanced safety on the roads.

## 2. Related Work

### 2.1. State-Wise Safe Reinforcement Learning

State-Wise Safe Reinforcement Learning (SRL) is an advanced paradigm within the field of reinforcement learning that emphasizes the enforcement of safety constraints at every step of the learning process. This is particularly crucial in applications where safety is of paramount importance, such as autonomous vehicle navigation and robotic manipulation.

In SRL, the notion of state-wise safety is pivotal. It requires that for any given state, the actions taken by the agent do not violate predefined safety criteria. These criteria are often encapsulated by a set of cost functions $C_{1},C_{2},\ldots ,C_{m}$, which quantify the safety of state–action transitions. An action *a* in state *s* is considered safe if it satisfies the constraints imposed by these cost functions.

Control Barrier Functions (CBFs) are mathematical tools derived from control theory that are used to define a region of the state space where the system is guaranteed to remain safe. The CBF-based methods in SRL leverage these functions to ensure that the agent’s actions maintain the system within a safe operating region.

The CBF-based SRL can be articulated through the following steps:

Safe Region Specification: Define a safe region ${\bar{\Pi }}_{C}$ in the state space using a Lyapunov-like energy function v:S→R. The safe region is characterized by the level set V(c)={s∈S∣v(s)≤c}, where *c* is a chosen threshold.

CBF Construction: Construct a $\mathrm{CBF}\tilde{h}:S\times A\to R$ that quantifies the distance to the boundary of the safe region. The CBF should satisfy $\tilde{h}\left(s,a\right)\geq 0$ for all (s,a) within the safe region.

Policy Design: Design a policy π that optimizes the expected cumulative reward while ensuring that the CBF remains positive, thus keeping the system within the safe region.

The barrier function serves as a formal safety certificate associated with a control policy, guaranteeing the state-wise safety of a dynamical system [21,22]. Classical control theory often relaxes the stringent conditions of the barrier function into optimization formulations like linear programs [23,24] and quadratic programs [25,26].

Recent research has explored the joint learning of control policies and neural barrier functions to optimize state-wise safety constraints in reinforcement learning [27,28,29]. In the context of autonomous driving, ShieldNN [30] leverages CBF to design a safety filter neural network, providing safety assurances for environments with known bicycle dynamics models. Ref. [31] adopts an architecture akin to ShieldNN, employing a safety filter to furnish demonstrations for RL algorithms, consequently enhancing sample efficiency.

However, a major challenge for these approaches is their limited scalability to higher-dimensional systems, particularly those with pixel observations.

### 2.2. Latent Dynamic Models

Latent dynamic models [32,33,34] represent a class of methods used for modeling time-series data with widespread applications in reinforcement learning (RL). These models are typically employed to capture the relationship between the hidden states of a system and the observed data, which is a crucial aspect in RL tasks as they assist agents in understanding the environment and making appropriate decisions.

In reinforcement learning, latent dynamic models are utilized to model the dynamic changes and hidden states of the environment. These models are often based on probabilistic frameworks, employing methods such as Bayesian inference or maximum likelihood estimation to learn model parameters and hidden states [35,36].

Mathematically, latent dynamic models can be represented as follows:

(1) $$\mathrm{State}\;\mathrm{Transition}\;\mathrm{Equation}:\;s_{t+1}=f\left(s_{t},a_{t},\epsilon_{t}\right)$$

This equation describes the process of state transition in the system, where $s_{t}$ represents the hidden state at time *t*, $a_{t}$ denotes the action chosen by the agent at time *t*, and $\epsilon_{t}$ represents environmental noise or randomness. The function *f* can be deterministic or incorporate some degree of randomness.

(2) $$\mathrm{Observation}\;\mathrm{Equation}:\;o_{t}=g\left(s_{t},\delta_{t}\right)$$

This equation describes the observation $o_{t}$ obtained when the state $s_{t}$ is observed, where *g* is a function and $\delta_{t}$ represents observation noise.

(3) $$\mathrm{Reward}\;\mathrm{Function}:\;r_{t}=R\left(s_{t},a_{t}\right)$$

This equation represents the immediate reward obtained by the agent when taking action $a_{t}$ in state $s_{t}$.

The objective of latent dynamic models is to infer the hidden state sequence $s_{1},s_{2},\ldots ,s_{T}$ from observed data $o_{1},o_{2},\ldots ,o_{T}$, action data $a_{1},a_{2},\ldots ,a_{T}$, and possible rewards $r_{1},r_{2},\ldots ,r_{T}$. This facilitates a better understanding of environmental dynamics and enables informed decision making.

These models are utilized to learn the dynamic model of the environment from observation data, enabling agents to predict the next state or observation. Additionally, they are employed in optimizing policies by integrating environmental dynamics modeling to maximize cumulative rewards [12,37,38]. Furthermore, latent dynamic models contribute to inferring the current hidden state of the environment based on observation data, thereby enhancing the agent’s understanding of the environment and facilitating decision-making [39,40]. In conclusion, latent dynamic models play a crucial role in reinforcement learning by assisting agents in comprehending complex environments and making effective decisions.

## 3. Problem Modeling

In the field of reinforcement learning (RL), an autonomous agent faces the challenge of interacting with an uncertain environment in a sequential manner to optimize a given utility signal. This interaction is typically modeled using a finite-horizon Markov Decision Process (MDP), which is represented by the tuple M∼(S,O,A,P,r,γ). In this formulation, $S\subset R^{n}\left(n\in Z+\right)$ represents a continuous state space, $A\subset R^{m}\left(m\in Z+\right)$ denotes the continuous action space, and the environment’s transition dynamics are governed by $s_{t+1}∼P\left(\cdot |s_{t},a_{t}\right)$, where $s_{t}\in S$ and $a_{t}\in A$. The observation space $O\subset R^{c_{o}\times h_{o}\times w_{o}}\left(c_{o},h_{o},w_{o}\in Z+\right)$ is derived from the state space and is captured by the agent’s camera module. The reward function is defined as r:S×A×S→R, and γ∈[0,1] represents the discount factor. In this setting, the agent’s control policy πθ generates actions $a_{t}$ based on observed images $o_{t}$, i.e., $a_{t}\in A∼\pi \left(\cdot |o_{t}\right)$, reflecting real-world scenarios where agents must act without direct access to the true state.

A crucial aspect of this MDP framework is the emphasis on state-wise safety, which is acknowledged by the presence of potentially hazardous states within a subset $S_{u}\subset R^{n}$. A safety violation occurs when the agent enters a state $s_{t}\in S_{u}$, which can be detected by a safety mechanism κ. As a result, the objective of state-wise safe RL with pixel observation is to optimize the control policy to maximize the expected cumulative discounted reward, which is subject to a constraint on the total number of safety violations:

(4) $$\max_{\theta }J\left(\pi_{\theta }\right)=E_{P\left(\cdot |s_{t},a_{t}\right)}\left[\sum_{t=0}^{T}\gamma^{t}r\left(s_{t},a_{t},s_{t+1}\right)\right],\;s.t.\;,\sum_{t=0}^{T}\kappa \left(s_{t}\right)\leq D,a_{t}∼\pi_{\theta }\left(\cdot |o_{t}\right).$$

Here, $\kappa (s_{t})\in 0,1$ serves as an indicator of safety violations, and D∈R represents the allotted budget for such violations. In practical, safety-critical systems, it is crucial to mitigate safety violations, ideally aiming for D→0 during the learning process.

This formulation is similar to the concept of constrained MDPs (CMDPs), where the agent must learn to navigate an environment while minimizing a safety cost, which is analogous to the scalar cost variable $c_{t}\in R$ in CMDPs. The agent’s policy $\pi^{*}$ is optimized to ensure that the expected sum of these costs remains below a predefined safety threshold d∈R, highlighting the dual objectives of maximizing rewards and satisfying safety constraints.

## 4. Methods

**Figure 1:**
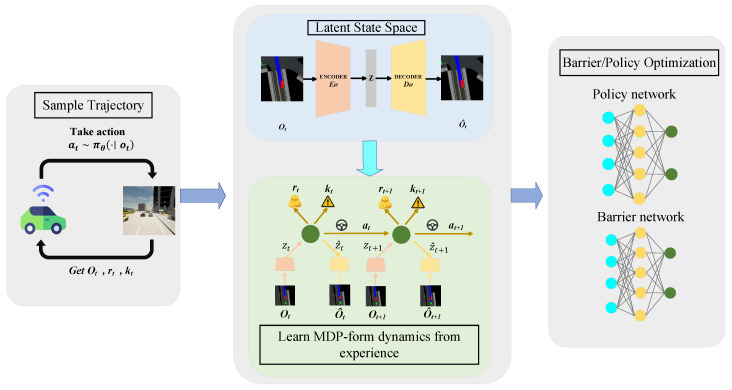
Overview of our joint learning framework for autonomous agents. The framework leverages real-world BEV data to learn a low-dimensional latent representation that captures the underlying dynamics of the environment using an MDP formulation. Subsequently, a latent barrier function is learned on top of this latent space to encode state-specific safety constraints.

We propose a novel framework for state-wise safe PPO with a latent state in the context of autonomous driving. Figure 1 depicts the high-level architecture, encompassing latent state modeling, barrier function learning, and policy optimization.

**Figure 2:**
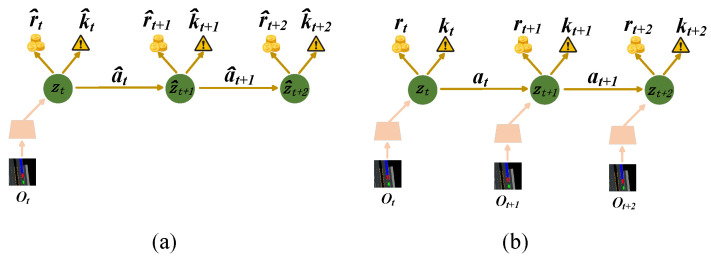
(**a**) In an imaginary environment, the agent forecasts state values and driving actions that maximize predicted future value. This is achieved by backpropagating gradients through simulated trajectories. (**b**) RL agent interacts with driving environment.

To mitigate the challenges associated with high-dimensional BEV (bird’s-eye view), we introduce a latent space representation. This is achieved by compressing the BEV image into a low-dimensional latent vector using a Variational Autoencoder (VAE)-like approach. We further learn latent dynamics within this space, enabling the model to capture temporal dependencies; this process is shown in Figure 2. By leveraging the power of learning, this approach can effectively handle the complexities of the driving environment, including non-smooth contacts and rich interaction dynamics. Crucially, a latent safety predictor is incorporated within the framework to identify unsafe regions in the latent space. As shown in Figure 2a, this learned safety information is then utilized to construct an MDP-like latent model, functioning as a generative model for producing synthetic training data. Consequently, the approach operates in a model-based fashion, minimizing interactions with the real environment and reducing the risk of safety violations during training.

Building upon the foundation of the latent dynamics model, a latent barrier-like function is introduced. This function encodes state-wise safety constraints within the latent space. Safety labels are generated from the learned latent safety predictor and used to train the barrier function on synthetic data. Notably, the training gradients from the barrier function can propagate back to the control policy, encouraging it to select safer actions. Concurrently, policy optimization is performed to maximize the total expected return in a model-based manner. Algorithm 1 summarizes the overall framework. Subsequent sections will delve deeper into each component.
**Algorithm 1** State-Wise Safe PPO with Latent Dynamics**Require:** Initial policy $\pi_{\theta }$, generated horizon *H*, action repeat *R*, collect interval *C*, batch size *B*, chunk length *L*, total episodes *E*, episode length *T***Ensure:** Policy $\pi_{\theta }$ with barrier-like function $B_{\theta }$ and the latent model with ϕ Initialize dataset D with random seed episodes Initialize models with parameters θ,ϕ, and ω **for** epoch =1,…,E **do**     **for** update step =1,…,C **do**         Sample batch of sequence chunks ${\left\{{\left(o_{t},a_{t},r_{t},\kappa_{t}\right)}_{t=k}^{L+k}\right\}}_{i=1}^{B}∼D$         Train latent model and calculate $L_{m}$ from Equation (5)         Update $\phi \leftarrow \phi +\varphi \nabla_{\phi }L_{m}$ // Update the latent model         Generate trajectories ${\left\{{\left(z_{t},a_{t},{\hat{r}}_{t},\hat{\kappa }\left(z_{t}\right)\right)}_{t=\tau }^{\tau +H}\right\}}_{i=1}^{B\times (L+k)}$ using current policy in latent space         Compute $L_{b}$ from Equation (9), $L_{total}$ from Equation (12)         Update $\theta \leftarrow \theta +\alpha \nabla_{\theta }L_{p}$         Update $\omega \leftarrow \omega +\alpha \nabla_{\omega }\sum \hat{E}t\left[{\left(v_{\omega }\left(z_{t}\right)-{\hat{V}}_{t}\right)}^{2}\right]$     **end for**     **for** i=1,…,T/R **do**         Compute $z_{t}$ and $a_{t}$ from latent model and $\pi_{\theta }$, add exploration noise on $a_{t}$         $r_{t},\kappa_{t+1},o_{t+1}\leftarrow$ env.step$\left(a_{t}\right)$     **end for**     Add the new trajectory to D **end for**


### 4.1. Input Representation

**Figure 3:**
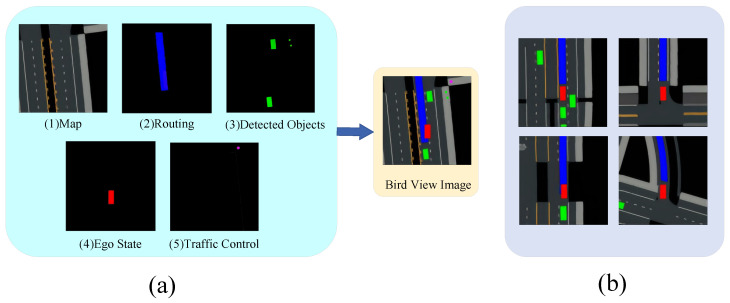
(**a**) Input representation of our framework. The bird’s-eye view observation combines information from the map, routing, detected objects, ego state, and traffic control. (**b**) BEV maps in different driving scenarios.

In autonomous driving systems, a BEV semantic mask is leveraged to provide a comprehensive overview of road conditions and nearby objects. Following similar approaches [11,12,20], we employ a mask structured as a 64 × 64 × 3 tensor, as visualized in Figure 3. The semantic mask encompasses five key elements:

1. Map: The tensor encompasses a map segment that portrays the road network’s layout. Drivable areas and lane markings are rendered in the map.
2. Routing: Information regarding the planned path, composed of waypoints determined by a route planner, is integrated into the mask. This information is represented as a bold blue line, guiding the autonomous vehicle along its designated route.
3. Detected Objects: The tensor includes bounding boxes representing detected surrounding road participants. These participants can include vehicles, bicycles, and pedestrians.
4. Ego State: The position and orientation of the autonomous vehicle itself are indicated by a red box within the tensor.
5. Traffic Control: Components that inform the vehicle of traffic rules, like traffic lights and stop signs, are depicted with varying levels of brightness to signal their status. Active stop signs and red traffic lights are shown with the brightest colors for visibility. Yellow lights use a medium brightness level, and green lights are displayed with the darkest shade.

This semantic mask simplifies the high-dimensional raw sensor data, distilling it into a format that retains the essential information required for the vehicle’s navigation and decision-making processes. By transforming complex visual and spatial data into this bird’s-eye view tensor, the system can efficiently process and act upon the information necessary for safe and effective autonomous driving.

### 4.2. Latent State Space with Latent Dynamics

In order to address the complexity of high-dimensional input data and mitigate the risk of overfitting during the reinforcement learning process, our methodology employs a dimensionality reduction strategy. We leverage a Variational Autoencoder (VAE) architecture to derive a low-dimensional latent representation of the environment. The VAE comprises an encoder network, denoted by $E_{\phi }$, to compress high-dimensional pixel observations O into a low-dimensional latent space Z, and a decoder network, denoted by $D_{\phi }$, which aims to reconstruct observations from the latent space. The encoder and decoder are parametrized by ϕ, which are optimized to minimize the loss function composed of various components:

(5) $$L_{m}=\sum_{t=0}^{T}\left(\mathrm{KL}\left(z_{t}∥E_{\phi }\left(o_{t}\right)\right)+\mathrm{MSE}\left({\hat{r}}_{t},r_{t}\right)+\mathrm{MSE}\left({\hat{\kappa }}_{t},\kappa_{t}\right)+\mathrm{MSE}\left({\hat{o}}_{t},o_{t}\right)\right)$$

In the above formulation, the Kullback–Leibler (KL) divergence quantifies the discrepancy between the probability distribution of the inferred latent states $z_{t}$ and the actual distribution of the states compressed from the real observations $E_{\phi }\left(o_{t}\right)$. This component is essential for refining our transition model T. The Mean Squared Error (MSE) terms are utilized to train the reward predictor and safety predictor as well as to capture the fidelity of the observation reconstruction from the latent space.

The encoder and decoder are Convolutional Neural Networks (CNNs) and transposed CNNs, respectively, while the reward and safety predictors are modeled using Deep Neural Networks (DNNs). The transition model T is designed as a Recurrent Neural Network (RNN), enabling the capture of temporal dependencies and dynamics. Our latent model structure builds upon the Recurrent State-Space Model (RSSM) proposed by [7], but it introduces a novel interpretation of the latent state space and incorporates a safety predictor.

Our approach transforms the environment’s Markov Decision Process (MDP) into an MDP-like latent model characterized by a low-dimensional latent space, as illustrated in Figure 1. The latent space $\left(Z,A,T,\hat{r},\hat{\kappa },\gamma \right)$ is designed to reflect the dynamics of the environment in a compressed manner while also integrating reward and safety signals. We posit the existence of a safety detector $\kappa \left(s_{t}\right)$, which may be an amalgamation of various sensor modalities. The purpose of the latent safety predictor $\hat{\kappa }\left(z_{t}\right)$ is to estimate this detector’s outputs such that potential safety violations within the latent space can be identified.

The transition model T allows us to emulate the environment’s dynamics within the latent space, providing Gaussian predictions for subsequent latent states based on current states and actions, that is, $z_{t+1}∼T\left(\cdot |z_{t},a_{t}\right)$. This latent model, which retains the same control policy as the actual MDP of the environment, can function as a generative model to synthesize data for training the control policy. By sampling latent trajectory data ${\left\{\left(z_{t},a_{t},{\hat{r}}_{t},\hat{\kappa }t\right)\right\}}_{t=0}^{T}$, the interaction with the real environment during training is minimized, thereby reducing exposure to unsafe conditions.

The training of the latent model utilizes trajectory chunks of time length *T* from the real environment MDP’s data buffer, adopting the loss function defined earlier. In doing so, the model learns to navigate transitions that may not be smooth, thereby addressing the challenges posed by the complex raw sensor data.

### 4.3. Control Barrier Functions as Safety Certificates

In the context of machine learning and the formulation of a safe latent state space, we establish a barrier-like function that serves as a demarcation between safe and unsafe states, which is guided by a safety predictor delineated within the latent model.

For a given policy $\pi_{\theta }$, the barrier-like function $B_{\theta }$ is defined in the latent state space, emphasizing the following safety conditions:

Safety Condition for Safe States: For all safe states $z_{s}\in Zs$, the barrier function $B\theta (z_{s})$ must exceed a positive threshold η, denoting the state’s location with in the safe region:

(6) $$B_{\theta }\left(z_{s}\right)>\eta$$

Continuity Condition for State Transitions: The model mandates that the barrier function’s value remains positive while transitioning from a current safe state $z_{t-1}$ to the next expected state $E(z_{t}|z_{t-1})$. This continuity condition is integral to ensuring that the barrier function’s value for the expected next state is greater than that of the current state, adjusted by a term involving α, which is a positive class-K function:

(7) $$B_{\theta }\left(E\left(z_{t}|z_{t-1}\right)\right)-B_{\theta }\left(z_{t-1}\right)+\alpha B_{\theta }\left(E\left(z_{t}|z_{t-1}\right)\right)>0$$

Safety Condition for Unsafe States: In contrast, for all unsafe states $z_{u}\in Zu$, the barrier function $B_{\theta }\left(z_{u}\right)$ should fall below the positive threshold η, signifying that the state is within the unsafe region:

(8) $$B_{\theta }\left(z_{u}\right)<\eta$$

Here, the latent states $z_{s}$ and $z_{u}$ are part of the subsets Zs and Zu of the latent space Z, respectively. The transition model $T(\cdot |z_{t-1},\pi_{\theta }\left(z_{t-1}\right))$ governs the distribution of $z_{t}$. The parameters θ encapsulate the characteristics of the barrier-like function and the policy network, reinforcing the safety framework within the latent space.

Within this framework, safe and unsafe latent states are distinguished by the learned safety predictor such that $\hat{\kappa }\left(z_{t}\right)=1$ for $z_{t}\in Z_{u}$, and $z_{t}\in Z_{s}$ otherwise. The essence of the latent barrier-like function is to provide a state-wise safety measure, ensuring that an agent remains in the safe subset by maintaining a positive barrier function value, as approximated by the second condition.

However, partial observability can lead to instances where the agent inadvertently enters unsafe regions. Partial observability in autonomous driving stems primarily from limitations in sensory technologies and environmental complexities that obstruct complete data acquisition. Sensors might fail to detect hidden or obscured hazards due to angle, distance, or adverse weather conditions. Furthermore, the unpredictable dynamics of road traffic, such as sudden stops or unexpected pedestrian movements, can go undetected until they pose immediate risks. These gaps in sensory information mean that the vehicle’s decision-making algorithms may not have access to all necessary data to make safe choices. Consequently, the system might make decisions based on incomplete or outdated information, thus inadvertently steering the vehicle into scenarios that increase the likelihood of accidents or safety breaches. This highlights the critical need for robust models that can infer the full scope of the environment from partial inputs and predict potential hazards with high accuracy. The barrier-like function aids in guiding the agent back to safety by leveraging the difference in the consecutive state function values, which is in contrast to the cumulative cost minimization focus of CMDP approaches.

Our study incorporates a stochastic component into the mean of the transition model’s distribution, represented by $z_{t}$, which is a common practice in model-based approaches. Our experimental results indicate that neglecting this randomness leads to a decline in the reconstruction quality and policy performance due to a deterministic path from the encoder output to the decoder input.

To operationalize the latent barrier-like function, we employ a dense neural network and derive a loss vector as inspired by prior work [17,41]:

(9) $$L_{b}=\left[\mathrm{ReLU}\left(\eta -B_{\theta }\left(z_{s}\right)\right),\mathrm{ReLU}\left(\eta -B_{\theta }\left(z_{t},z_{t-1}\right)\right),\mathrm{ReLU}\left(\eta +B_{\theta }\left(z_{u}\right)\right)\right]$$

This loss vector penalizes non-positive safe states, enforces the positivity of the time derivative condition, and penalizes non-negative unsafe states. A small positive learning margin η is introduced to facilitate optimization. Due to the inherent partial observability of the system, the formulation can only promote forward invariance without absolute guarantees.

In this revised equation, $B_{\theta }\left(z_{s}\right)$ represents the evaluation function for safe states, assessing the safety of state $z_{s}$. $B_{\theta }\left(z_{t},z_{t-1}\right)$ is the positive evaluation of the time derivative condition, reflecting whether the transition from state $z_{t-1}$ to $z_{t}$ adheres to the requirements for maintaining state safety. This could incorporate $B_{\theta }\left(z_{t}\right)-B_{\theta }\left(z_{t-1}\right)+\alpha \left(B_{\theta }\left(z_{t}\right)\right)$, which is now explicitly framed within the derivative evaluation context. $B_{\theta }\left(z_{u}\right)$ denotes the evaluation function for unsafe states, measuring the degree of unsafety for state $z_{u}$.

### 4.4. State-Wise Safe PPO

To optimize the total rewards while considering state-wise safety, we formulate an actor–critic approach with barrier-like function learning in the loop within the latent model by using the trajectories $\left\{z_{t},a_{t},{\hat{r}}_{t},{\hat{\kappa }}_{t}\right\}$ generated by the latent model. With the encoder network embedded inside, the policy network $\pi_{\theta }\left(\cdot |E\phi \left(o_{t}\right)\right)$ (or equivalently $\pi \theta \left(\cdot |z_{t}\right),z_{t}\approx E\phi \left(o_{t}\right)$) outputs action $a_{t}$ in a Gaussian distribution, which is randomly sampled for training and provides a mean action value for evaluation.

The value (critic) function of RL can be expressed as

(10) $$v_{\omega }\left(z_{t}\right)=E_{\pi \theta \left(\cdot |z_{t}\right)}\left[\sum_{t=\tau }^{\tau +T}\gamma^{t}\hat{r}t\right]$$

where $v_{\omega }\left(z_{t}\right)$ represents the expected cumulative discounted reward starting from state $z_{t}$ and following policy $\pi_{\theta }$. The discount factor γ is used to balance the importance of immediate and future rewards.

In the Proximal Policy Optimization (PPO) algorithm, the optimization objective is modified to incorporate a clipped surrogate objective, which can be expressed as shown below:

(11) $$L_{CLIP}\left(\theta \right)=\hat{E}t\left[\min\left(r_{t}\left(\theta \right)\hat{A}t,\mathrm{clip}\left(r_{t}\left(\theta \right),1-\epsilon ,1+\epsilon \right)\hat{A}t\right)\right]$$

where $r_{t}\left(\theta \right)=\frac{\pi_{\theta }\left(a_{t}|s_{t}\right)}{\pi_{\theta \mathrm{old}}\left(a_{t}|s_{t}\right)}$ is the probability ratio between the current policy πθ and the previous policy πθold. The advantage function ${\hat{A}}_{t}$ estimates how much better the current action is compared to the average action in state $s_{t}$. The clip function limits the ratio within the range [1−ϵ,1+ϵ] to prevent excessive policy updates.

Combining the clipped surrogate objective with the barrier loss function as a regularization term for safety, the overall optimization objective becomes

(12) $$L_{total}\left(\theta \right)=L_{CLIP}\left(\theta \right)+\beta^{T}L_{b}$$

where β is a hyperparameter controlling the trade-off between reward optimization and safety constraint satisfaction, and $L_{b}$ is the barrier loss function that penalizes unsafe actions.

The critic network $v_{\omega }\left(z_{t}\right)$ is optimized by minimizing the squared error between the estimated value and the target value obtained through Monte Carlo estimation:

(13) $$L_{V}\left(\omega \right)=\hat{E}t\left[{\left(v_{\omega }\left(z_{t}\right)-{\hat{V}}_{t}\right)}^{2}\right]$$

where ${\hat{V}}_{t}=\frac{1}{N}\sum_{i=1}^{N}\left[\sum_{\tau =0}^{T}\gamma^{\tau }{\hat{r}}_{\tau }+\gamma^{t+T}v_{\omega }\left(z_{t+T}\right)\right]$ is the target value estimated using the discounted sum of rewards and the value of the state at the end of the trajectory.

To estimate the advantage function, we use the Generalized Advantage Estimation (GAE):

(14) $$\hat{A}t=\sum_{l=0}^{T-1}{\left(\gamma \lambda \right)}^{l}\delta_{t+l}$$

where $\delta_{t}=\hat{r}t+\gamma v\omega \left(z_{t+1}\right)-v_{\omega }\left(z_{t}\right)$ is the temporal difference error, and λ is a hyperparameter controlling the trade-off between bias and variance in the advantage estimation.

The optimization process involves sampling a batch of trajectories $\left\{z_{t},a_{t},\hat{r}t,\hat{\kappa }t\right\}$ using the current policy $\pi_{\theta \mathrm{old}}$, estimating the advantage function ${\hat{A}}_{t}$, optimizing the critic network by minimizing $L_{V}\left(\omega \right)$, and optimizing the actor network by maximizing $L_{total}\left(\theta \right)$ using stochastic gradient ascent. This process is repeated until convergence, allowing the agent to learn a policy that maximizes the expected cumulative discounted reward while satisfying safety constraints.

## 5. Experiments

### 5.1. Environmental Setup

We conducted training and evaluation of our proposed method using the CARLA simulatorr [42]. CARLA is a high-fidelity, open-source simulation platform specifically designed for autonomous driving research. It excels not only in simulating the driving environment and vehicle dynamics but also leverages rendering and ray-casting techniques to generate realistic sensor data inputs, including camera RGB images and LiDAR point clouds.

**Figure 4:**
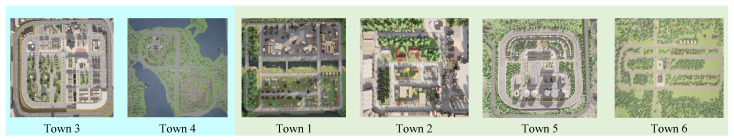
CARLA encompasses a diverse set of road networks within its simulated environments, ranging from Town01 to Town06. These networks offer a variety of driving scenarios, from simple layouts to complex environments with challenging features such as roundabouts, tunnels, and infinite highways. To train and evaluate our models, we utilized Town03 and Town04 as the training set, while the remaining towns were used as the test set.

To comprehensively assess the performance of our algorithm, we employed six diverse maps within CARLA, encompassing Town01 to Town06 (as depicted in Figure 4). The towns vary in complexity and features. Town01 is a simple town with a river and bridges. Town02 is similarly uncomplicated, featuring a blend of residential and commercial structures. Town04 stands out with its mountainous surroundings and a distinctive infinite highway. Town05 is characterized by a grid layout, intersections, and a bridge. Town06 encompasses lengthy multi-lane highways with numerous entrances and exits. Lastly, Town03 is the most complex, offering challenges such as a 5-lane junction, a roundabout, uneven terrain, a tunnel, and other obstacles.

To guarantee the robust generalization of our autonomous driving model, we opted to train it initially on Town03 and Town04. Town03 exemplifies urban complexity, featuring a downtown area with a central roundabout, diverse intersections, and an underpass, which necessitates the development of advanced navigation capabilities. Furthermore, its architectural variety, encompassing parks and commercial zones, exposes the model to a broad spectrum of urban conditions. In contrast, Town04, nestled against a mountainous backdrop, presents a natural landscape that challenges the model’s adaptability to suburban environments and high-speed highway scenarios. The incorporation of both random and fixed vehicle scenarios within these towns, following CARLA’s randomization protocols, ensures comprehensive training that equips the model to handle both urban and suburban complexities, ultimately enhancing its generalizability.

### 5.2. Network Architecture

In order to provide a more comprehensive introduction to the employed models, we delve into the specifics of their network architectures.

- Variational Autoencoder (VAE): This model consists of an encoder and a decoder, each encompassing 4 convolutional layers. The encoder uses 3×3 kernels with increasing channels of 32, 64, 128 and 256, utilizing a stride of 2 to effectively reduce the dimensionality of input BEV images. These processed data are then compressed into a latent space layer of size 1024 and connected to the output of the last convolutional layer. The decoder mirrors this architecture but uses transposed convolutional layers to upscale the latent vector back to the original dimensions, gradually reducing channels from 256 to 3, with each layer employing ReLU activation to ensure data integrity through non-linearity.
- Transition Model: This model is designed for handling sequential data and predicting future states, starting with a fully connected layer that merges input state and action vectors, followed by a GRU cell maintaining a belief state sized at 200. This model splits into two pathways post-GRU, each consisting of two fully connected layers where the first layer outputs an intermediate representation of size 256 and the second computes the means and standard deviations of the state distributions, which are both sized at 30.
- Reward Predictor: The reward predictor begins with a fully connected layer taking a concatenated vector of belief and state (size 230), followed by two additional layers, each with 128 channels equipped with ReLU activations, culminating in an output layer that yields a single reward value.
- Safety Predictor: This model starts with a concatenated input of belief and state processed through three fully connected layers, each also with 128 channels and ReLU activations. This model outputs a single value that represents the safety cost associated with different states and actions, which is crucial for maintaining adherence to safety constraints within the operational environment.
- Control Barrier Function: This module consists of a neural network designed to assess and enforce safety constraints, focusing on the latent representations of states. It is structured with three fully connected layers. The network accepts an input of the latent state, typically of size 1024 from the encoded state representation, and processes this through its layers to ultimately output a single value. This output quantifies the safety risk associated with the current state, guiding the agent’s actions to prevent transitions to potentially hazardous states.
- Soft Actor–Critic (SAC) [3,12]: This is a key reinforcement learning method that employs a comprehensive neural network architecture that includes two Q-networks, one value network, and a stochastic policy network. Both the Q-networks and the value network are structured similarly, featuring visual encoding layers followed by five dense layers with hidden units decreasing from 256 to 32, each ending with a single output unit to predict the value of actions given state inputs. Distinctively, the policy network follows the same initial setup but diverges at the final layer, branching into two separate outputs to represent the mean and variance of the action distribution. This bifurcation allows the policy to probabilistically explore actions, enhancing the algorithm’s capacity for both exploration and exploitation. All components within the SAC are optimized using the Adam optimizer with a learning rate of $3\times 10^{-4}$, ensuring consistent and effective learning across diverse scenarios encountered by the agent. This structured network setup enables SAC to adeptly balance decision-making strategies under the complex uncertainties of reinforcement learning environments.
- Proximal Policy Optimization (PPO) [2]: Referred to as Roach [20], the network’s architecture is meticulously designed to handle the complexities of autonomous navigation, comprising an input encoding phase and a subsequent decision-making phase. During the encoding phase, the input is intricately processed through six convolutional layers, which adeptly extract relevant spatial features. The decision-making phase merges the encoded information into a latent feature vector through two additional fully connected layers. This latent representation is then leveraged by both the policy and value networks, which are each composed of two fully connected hidden layers that lead to their specific output layers. The policy network is distinctive for its output-a beta distribution over the continuous action space $a\in {\left[-1,1\right]}^{2}$, delineating the distribution of steering and acceleration actions. Meanwhile, the value network provides a scalar estimation *v* of the expected return, integrating the current state and action’s potential future rewards.

### 5.3. Cost Functions

In the domain of autonomous driving, the conception of cost functions ${\hat{\kappa }}_{t}\left(z_{t}\right)\in \left\{0,1\right\}$ is instrumental in embedding the essential facets of driving safety into the vehicle’s decision-making processes. These cost functions are foundational to the development of a safety reinforcement learning framework, as they quantify various dimensions of safety risks and enforce regulations. By integrating these costs, we can steer the learning algorithms toward strategies that not only aim for efficiency but also adhere strictly to safety protocols. The significance of defining these costs is paramount, as they serve a dual purpose: to deter unsafe actions by imposing penalties and to guide the autonomous system toward a ’safe status’ where the likelihood of hazardous events is minimized. Informed by the valuable insights gleaned from these research works [43,44,45], we propose the following cost functions pertaining to the “safe state”:

1. Collision Cost: This cost is applied when the autonomous vehicle engages in a collision with other vehicles, pedestrians, or static obstacles. The Collision Cost is meticulously designed to impart penalties for such incidents, promoting the reinforcement learning algorithm to favor safe navigation tactics.
2. Traffic Violation Cost: This cost captures the instances when the autonomous vehicle commits infractions of traffic laws, such as exceeding speed limits or failing to stop at red signals. The Traffic Violation Cost plays a crucial role in ensuring the vehicle’s compliance with traffic rules, thereby bolstering road safety.
3. Road Markings Cost: Compliance with road markings is essential for maintaining orderly traffic flow. This cost penalizes deviations such as crossing solid lines or entering forbidden zones. The Road Markings Cost serves to guarantee that the vehicle’s path remains within legal boundaries and respects road etiquette.
4. Tailgating Cost: Maintaining a safe following distance from leading vehicles is critical to averting rear-end collisions. The Tailgating Cost is employed to deter the autonomous vehicle from maintaining an excessively close proximity to the vehicle ahead, thus diminishing the chances of accidents in scenarios where the lead vehicle decelerates or stops abruptly.

These cost functions are critical to shaping a robust control architecture for autonomous vehicles that prioritizes safety. They capture the multi-faceted nature of driving safety and are integral in directing the learning algorithm toward conduct that satisfies both efficiency and safety regulations. Embedding these costs within the reinforcement learning framework is fundamental to training the autonomous system to balance task accomplishment with a safety-first approach.

### 5.4. Rewards Design

The definition of reward that we employ is very similar to those works [11,13]. The reward function considers the agent’s velocity, position, rotation, and penalizes idleness. Here is the modified formula description for the reward function.

**Figure 5:**
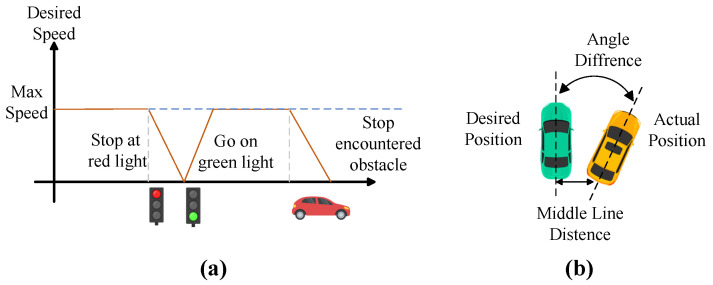
(**a**) In response to the surrounding environment, the agent dynamically adjusts its desired speed. This desired speed decreases as the vehicle approaches a red light, returning to its maximum value when the light turns green. Similarly, the desired speed decreases again when approaching an obstacle. The reward function is maximized when the vehicle’s actual speed aligns with the desired speed. (**b**) For calculating the position and angular reward components, the system considers the distance and angular difference between the ideal position, designated in green, and the current position of the agent, represented in yellow.

1. $R_{\mathrm{speed}}$: expected velocity reward, illustrated on Figure 5a.
  (15) $$R_{\mathrm{speed}\;}=\left\{\begin{array}{cc} 1 & \;\mathrm{if}\;v_{\mathrm{agent}\;}=v_{\mathrm{desired}\;}\\ \frac{1}{1+\alpha \cdot \left(\left|v_{\mathrm{agent}\;}-\;v_{\mathrm{desired}}\right|\right)} & \;\mathrm{otherwise}\; \end{array}\right.$$
  where $v_{\mathrm{agent}}$ is the actual velocity of the agent, $v_{\mathrm{desired}}$ is the desired velocity, and α is a positive constant that controls the penalty for deviation from the desired velocity.
2. $R_{\mathrm{position}}$: expected position reward.
  (16) $$R_{\mathrm{position}\;}=\left\{\begin{array}{cc} 0 & \;\mathrm{if}\;d_{\mathrm{lane}}=0\\ -1 & \;\mathrm{if}\;d_{\mathrm{lane}\;}>D_{\max}\\ \frac{D_{\max}}{d_{\mathrm{lane}\;}} & \;\mathrm{otherwise}\; \end{array}\right.$$
  This reward is inversely proportional to the agent’s lateral displacement from the lane center (calculated using predefined waypoints). This reward peaks at 0 when perfectly centered and linearly decreases to −1 at the maximum allowed lateral deviation, $D_{\max}$ (set to 2 m, corresponding to the lane width). Exceeding $D_{\max}$ triggers episode termination. Additionally, collisions, red light violations, and unexplained stalling (not due to obstacles or traffic signals) incur a −1 penalty and terminate the episode.
3. $R_{\mathrm{rotation}}$: expected rotation reward.
  (17) $$R_{\mathrm{rotation}\;}=\frac{1}{1+\beta \cdot (\Delta \theta )}$$
  where Δθ is the angular difference between the agent’s orientation and the expected direction of the nearest path point, and β is a positive constant that controls the penalty for angular deviation.The position reward and rotation reward are explained in detail in Figure 5b.
4. $R_{\mathrm{stationary}}$: stationary penalty.
  (18) $$R_{\mathrm{stationary}\;}=\left\{\begin{array}{cc} 0 & \;\mathrm{if}\;v_{\mathrm{agent}\;}>v_{\min}\\ -c & \;\mathrm{otherwise}\; \end{array}\right.$$
  where $v_{\min}$ is the set minimum velocity threshold to determine if the agent is stationary, and *c* is the constant term for stationary penalty.

The total reward ($R_{\mathrm{total}}$) can be a weighted sum of these four rewards; weights can be adjusted according to the specific problem:

(19) $$R_{\mathrm{total}\;}=w_{\mathrm{speed}\;}\cdot R_{\mathrm{speed}\;}+w_{\mathrm{position}\;}\cdot R_{\mathrm{position}\;}+w_{\mathrm{rotation}\;}\cdot R_{\mathrm{rotation}\;}-c\cdot R_{\mathrm{stationary}\;}$$

where $w_{\mathrm{speed}}$, $w_{\mathrm{position}}$, and $w_{\mathrm{rotation}}$ are the corresponding weights for the rewards, and $R_{\mathrm{stationary}}$ is an indicator that is 1 when the agent is stationary and 0 otherwise.

## 6. Results

To comprehensively evaluate the performance of our autonomous driving system, we performed extensive comparisons with existing methods, including PPO [2], SAC [3], and Roach PPO [20]. PPO and SAC are well-established deep reinforcement learning algorithms. Roach PPO builds upon PPO by representing the action space with a beta distribution and introducing a novel exploration loss function to enhance sample efficiency and problem quality, achieving promising results on the CARLA benchmark. This algorithm can serve as the baseline for our experiments, and its performance on autonomous driving tasks is primarily evaluated through comparative analysis.

We introduce Latent SW-PPO, which is a novel algorithm that integrates a latent model with a state-wise safe Proximal Policy Optimization (PPO) algorithm. To assess the independent contributions of each component, we conducted ablation studies. These studies employed two variants: Latent PPO, which utilizes only the latent model without the SRL constraint satisfaction function, effectively reducing the reinforcement learning component to a standard PPO algorithm; and SW-PPO, which utilizes the state-wise reinforcement learning CBF but omits the latent model, resulting in a conventional safe reinforcement learning algorithm. Given the shared foundation of reinforcement learning for all methods, we first evaluated their reward and cost trajectories during the training process. Subsequently, we assessed their performance in specific autonomous driving scenarios. This two-pronged approach facilitates a comprehensive and holistic evaluation of the algorithms.

### 6.1. Evaluating Reward and Safety

In conventional reinforcement learning, the performance of an algorithm is typically evaluated based on its reward. The reward function defines the immediate feedback that an agent receives for taking a specific action in a given state. The agent’s goal is to learn a policy that maximizes its cumulative reward over time. While this approach works well in many problems, it may be insufficient in scenarios involving safety-critical operations where relying solely on reward functions can be risky.

In safe reinforcement learning, the notion of cost becomes crucial. By introducing a cost function, safe reinforcement learning emphasizes not only maximizing reward but also maintaining safety during exploration and exploitation. This dual-objective optimization framework allows algorithms to pursue performance while ensuring the safety of their behavior, which is critical for safety-critical applications. In this way, safe reinforcement learning enables the development of intelligent systems that are both efficient and safe.

In safe reinforcement learning, AverageEpCost (Average Episode Cost) and CostRate [19] are two key metrics used to evaluate the performance of algorithms on safety-critical exploration problems.

AverageEpCost measures the average accumulated cost over a single episode, quantifying the agent’s total cost of interacting with unsafe elements during a complete episode. Assuming there are many time steps in an episode, and each time step t incurs a cost signal ${\hat{\kappa }}_{t}\left(z_{t}\right)$ from the agent’s interaction with unsafe elements in the environment, AverageEpCost can be calculated using the following formula:

(20) $$\mathrm{AverageEpCost}=\frac{1}{T}\sum_{t=1}^{T}{\hat{\kappa }}_{t}\left(z_{t}\right)$$

where *T* is the total number of time steps in the episode.

CostRate represents the average cost per time step during the agent’s training process, providing a more fine-grained measure of the safety of the agent’s behavior during training. CostRate is calculated by accumulating the cost signals from each time step in all episodes and then dividing by the total number of environment interaction steps. If there are *N* episodes, each with $T_{i}$ time steps, CostRate can be calculated using the following formula:

(21) $$\mathrm{Cos}\mathrm{tRate}=\frac{1}{I}\sum_{i=1}^{N}\sum_{t=1}^{T_{i}}{\hat{\kappa }}_{t}\left(z_{t}\right)$$

where *N* is the number of episodes, $T_{i}$ is the number of time steps in the *i* episode, and *I* is the total number of interaction steps across all episodes.

AverageEpCost emphasizes the model’s ability to interact safely in a single trial, while CostRate provides a more comprehensive view of the model’s safety and robustness over the long-term learning process. These metrics provide important tools for evaluating and comparing the safety of different algorithms.

#### 6.1.1. Evaluating Reward

**Figure 6:**
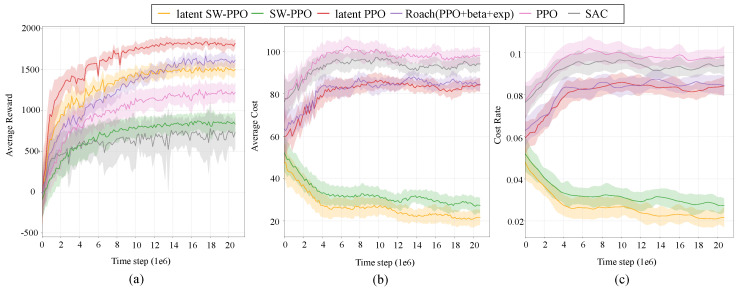
(**a**) Learning curves of different RL approaches. The shaded region represents half a standard deviation of returns over 1k evaluation steps. Curves are smoothed for visual clarity. (**b**) Cost curves of different RL approaches. Safe RL maintains a relatively low cost level. (**c**) Cost rate curves of different RL approaches.

As shown in the Figure 6a, the Latent PPO algorithm achieves the highest reward compared to other algorithms, and its slope indicates a rapid convergence speed. Latent SW-PPO also exhibits a relatively fast convergence speed despite not achieving the highest reward. Both of these algorithms begin to converge at approximately 10 M steps, while Roach and SW-PPO require nearly 20 M steps to converge gradually.

This demonstrates that by adopting a latent state space and generating synthetic data in the latent space, the algorithm can be trained without direct interaction with the real environment. This approach reduces the time and resources required for environmental interaction, thereby improving sample efficiency [11,12].

A comparison of the rewards of Latent PPO and PPO algorithms reveals that PPO converges prematurely with a low reward value, which is similar to the SAC algorithm. This suggests that conventional reinforcement learning algorithms still have limited capabilities in handling high-dimensional information.

The dimensionality reduction performed by the latent state space helps to alleviate the computational burden of directly processing the original high-dimensional data while preserving sufficient information for effective decision making. Dimensionality reduction can improve the learning efficiency of the algorithm by reducing the amount of data that needs to be processed, and it can also potentially help to avoid the “curse of dimensionality”.

We observed that the rewards obtained by the safe reinforcement learning algorithms SW-PPO and Latent SW-PPO are not as high as those of the traditional reinforcement learning algorithms Latent PPO and Roach. This is because conventional reinforcement learning algorithms can achieve high rewards by taking unsafe actions, which can also lead to higher costs. On the other hand, safe reinforcement learning algorithms will obtain lower rewards but can also control the cost within the desired range [19,46]. We will discuss the cost in more detail in the next subsection.

#### 6.1.2. Evaluating Safety

Figure 6b,c shows the AverageEpCost and CostRate of our approach and other algorithms. In principle, model-free reinforcement learning algorithms lead to more safety violations. As can be seen from the figure, the SW-PPO and Latent SW-PPO algorithms maintain AverageEpCost and CostRate at relatively low levels. These results affirm the advantages of our latent barrier-like function learning for encoding state-wise safety constraints. Additionally, Latent SW-PPO converges faster than SW-PPO. This is because during training, our latent model quickly identifies and captures the majority of unsafe latent states through supervised learning. With more interactions, the latent barrier-like encoding of hard state-wise safety constraints progressively forces the agent to take safer actions, leading to a lower Cost Return.

In autonomous driving, safety violations are inevitable. Considering vehicle dynamics and the unpredictability of the future environment, achieving zero violations is fundamentally a difficult problem to solve. Additionally, due to learning errors, our latent model may not always accurately distinguish between safe and unsafe images, which can lead to safety violations.

### 6.2. Driving Performance

In this section, we then compare the driving performance of our proposed algorithm against several methods. Finally, we conduct ablation studies to analyze the significance of various components within our approach.

#### 6.2.1. Metrics

The following outlines the metrics employed to evaluate the driving behavior of each agent in the Carla Leaderboard [47], which is a public leaderboard that ranks agents based on their performance in the Carla simulation environment. These metrics collectively provide a comprehensive understanding of an agent’s performance across various aspects of autonomous driving, including safety, efficiency, comfort, and compliance with rules.

1. Route Completion (RC): This metric is defined as the percentage of the route distance successfully completed by the agent on the $i^{th}$ route, which is symbolized by $R_{i}$. The average Route Completion across *N* routes is calculated as
  (22) $$RC=\frac{1}{N}\sum_{i}^{N}R_{i}*100\%$$
  It should be noted that if an agent deviates from the designated route lanes for a certain percentage of the route, the Route Completion is adjusted downwards by a factor of (−1% off route distance).
2. Infraction Score (IS):The Infraction Score is a composite measure that captures the cumulative impact of various infractions through a geometric series. Each type of infraction *j* has a corresponding penalty coefficient $p^{j}$ that is applied to the agent’s score for each infraction incurred during the route. The penalty coefficients are predefined: 0.50 for collision with a pedestrian, 0.60 for collision with a vehicle, 0.65 for collision with static objects, and 0.70 for running a red light. As our analysis indicates no instances of stop sign violations, this infraction type has been excluded. The Infraction Score for an agent starts at an ideal score of 1.0 and is reduced by the respective penalty coefficient for each infraction committed, as follows:
  (23) $$\;\mathrm{IS}\;=\prod_{j=\;\mathrm{Ped},\;\mathrm{Veh},\;\mathrm{Stat},\;\mathrm{Red}}{\left(p^{j}\right)}^{\#\;{\mathrm{infractions}}_{j}}$$
3. Driving Score (DS): The Driving Score is a weighted average that combines Route Completion with an infraction multiplier $P_{i}$. This represents the main metric on the leaderboard and is calculated for each route as follows:
  (24) $$DS=\frac{1}{N}\sum_{i}^{N}R_{i}P_{i}$$
4. Infractions per km: This metric accounts for the total number of infractions, which is normalized by the total number of kilometers driven. It should be noted that this metric is not a singular metric but rather a collection of metrics that share the same definition.The infractions included in this measure are collisions with pedestrians, vehicles, static objects; running red lights; off-road infractions; route deviations; timeouts; and vehicle blockages. The number of infractions per kilometer is computed as follows:
  (25) $$I/K=\frac{\sum_{i-1}^{N}I_{i}}{\sum_{i-1}^{N}K_{i}}$$
  here, $I_{i}$ represent the number of infractions for the *i*-th trip, $K_{i}$ represents the distance (in kilometers) driven for the *i*-th route, and *N* is the total number of trips. For off-road infractions, the total kilometers driven off-road is considered instead of the infraction count, and this metric is presented as a percentage by multiplying it by 100.

#### 6.2.2. Performance and Ablation

We first trained all models in Town03 and Town04; then, we evaluated them in Town01, Town02, Town05, and Town06, respectively. In each town, we generated 50 episodes with an average route length of 1.5 km, which is close to the average route length of the official leaderboard at 1.7 km. In each episode, the scene, driving route, and environmental vehicles were randomly generated to ensure the complexity and diversity of the evaluation. We ensured that the diversity and richness of the test data were comparable to those of the leaderboard and other algorithms. We recorded the performance of all models on the entire test set and on different towns. Table 1 shows the performance of all models in the test environment.

As can be seen from Table 1, Latent SW-PPO achieved the best results. It had the highest DS score, which was 60% higher than the Roach PPO algorithm. It also had the best path completion rate (RC) and safety index (IS) among all models. In terms of safety, both SW-PPO and Latent SW-PPO achieved higher IS scores than other algorithms. This confirms the advantages of our latent barrier-like function learning for encoding state-wise safety constraints over the CMDP formulation in the baselines. This conclusion is consistent with the conclusion that the cost changes during the reinforcement learning process.

Compared with SW-PPO, Latent SW-PPO uses a latent state space, which significantly improves the performance of the model. RC was improved by 35% and IS was improved by 21%. This indicates that latent dynamics enhances the understanding and prediction accuracy of complex environmental dynamics while improving data efficiency and model generalization ability. This allows the model to learn better with the same dataset.

The Roach PPO algorithm also achieved good results, even surpassing SW-PPO in path completion rate (RC). However, its safety score (IS) was lower. The Latent PPO algorithm also had similar problems. This indicates that without safety constraints, Roach PPO and Latent PPO may tend to violate rules in order to complete the driving task. This is consistent with the conclusion that reinforcement learning tends to make unsafe actions in order to pursue higher rewards during training. In our experiments, we also found that when the traffic scene is extremely congested, the Roach PPO algorithm will collide with cars or people in order to complete the task, while SW-PPO and Latent SW-PPO will choose to wait for the traffic congestion, which will result in the task not being completed and ending. This is the reason why SW-PPO’s path completion rate is lower than Roach PPO’s.

Experiments in the CARLA simulator demonstrate the importance of latent dynamics and safety constraints in reinforcement learning-based driving systems. The introduction of latent variable dynamics modeling and safety constraint mechanisms is valuable in the research of reinforcement learning driving systems. These mechanisms enable autonomous driving algorithms to learn safer and more intelligent strategies in complex environments.

#### 6.2.3. Evaluate Generalization

In this section, we analyze the generalization ability of our proposed model and compare its performance with other algorithms under different scenarios.We use three main metrics for evaluation: Driving Score (DS), Route Complete (RC), and Infraction Score (IS). We exclude SAC and PPO due to their poor performance and focus on comparing our algorithm with Roach PPO.

Table 2 shows the performance of different models on the training set (Town03 and Town04), and Table 3 shows their performance on the test set (Town01, Town02, Town05 and Town06). Town03 is a city block scene with more complex road features, such as roundabouts and overpasses. Town04 is a small town with a relatively simple road structure. Therefore, all models achieve lower Driving Scores in Town03 than in Town04, indicating that the complexity of the scene affects model performance.

Table 1 shows the average results of all models on the test set. Compared to Table 2, the Driving Scores of all models decrease on the test set. This indicates that models still experience some difficulties in adapting when encountering new scenes, leading to lower Driving Scores.

From the insights presented in Table 3, it is evident that the complexity of the environment plays a crucial role in the generalization performance of autonomous driving models. In simpler settings, as seen in Town01 and Town02, the Driving Scores do not decline as sharply, with models like Latent PPO and Latent SW-PPO even demonstrating improved performance in Town02. However, in more complex scenarios, such as those found in Town05 and Town06—which feature highways, expansive multi-lane roads, and intricate intersections—the Driving Scores experience a marked drop, with the decrease being particularly notable in Town05. These trends underscore a consistent observation within the realm of deep learning algorithms that the ability to generalize is significantly influenced by environmental complexity.

We analyze the generalization ability of different models in new environments, emphasizing the impact of scene complexity. The Latent SW-PPO model shows a minor decline in Driving Score, from 51.62 to 48.22 (a 6.5% drop), when transitioning from training to the test set. In contrast, Roach PPO’s score falls from 34.40 to 24.54, which is a significant 25.7% reduction.

**Figure 7:**
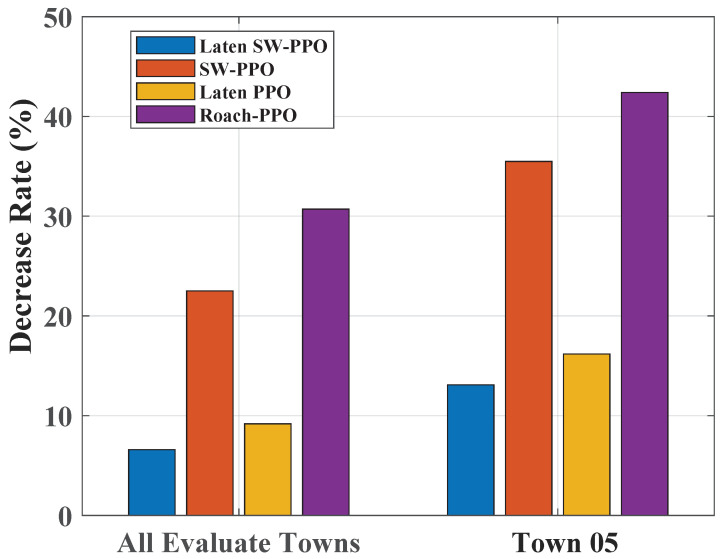
Decrease rate of driving score on the test set. The bar chart on the left represents the reduction rate for each model on the entire test set. On the right, the bar chart details the rate of decline specifically in Town05, which is identified as the test town experiencing the most significant reduction in driving scores.

The divergence in performance is more pronounced in complex scenarios, such as Town05. Here, Latent SW-PPO’s score drops by 13%, while Roach PPO’s plummets by 42.3%. Figure 7 illustrates the Driving Score drop, indicating that the models with latent state space, Latent PPO, and Latent SW-PPO experience a much smaller reduction compared to SW-PPO and Roach PPO.

The employment of latent state space in these models contributes to their improved generalization, which is evidenced by their robust performance against variations in input data. This capability enhances the models’ predictive and decision-making abilities in unfamiliar environments.

#### 6.2.4. Infraction Analysis

Through the previous analysis, our model has achieved good results in various metrics on the CARLA test. However, we still found some problems in driving during the experiment, which can be used as the direction of improvement for future research work.

We found that when some pedestrians or obstacles are close to the agent, the agent under SW-PPO control will stop, but sometimes this distance is still relatively far, and there will be no collision. SW-PPO sometimes overemphasizes safety, which can lead to a lot of time consumption. This is the reason why SW-PPO Route Complete does not perform well in many scenarios. Latent SW-PPO improves this problem, but in some cases it still exists. Therefore, how to balance efficiency and safety is a research that needs to be considered in the future.

We observed the error cases of Latent SW-PPO. In the case of heavy rain, Latent SW-PPO is more prone to errors. The main reason for this error is that the BEV map we currently use does not have climate information. In rainy weather in CARLA, the mechanical properties of the vehicle and the friction coefficient with the ground will change, which tends to make the agent adopt a more conservative strategy to ensure safety, such as slow speed or slow turning. However, we use the BEV map and do not perceive the change of environmental climate, so the collision occurs. Perception of the environment is the direction that needs to be improved in future work.

## 7. Conclusions

This research introduces a novel approach to safe reinforcement learning (SRL) for autonomous driving, combining latent space modeling with state-wise safety constraints. Our framework addresses the critical challenge of ensuring safety while optimizing performance in complex environments. By utilizing variational autoencoders, we have developed a latent space representation that enhances sample efficiency and reduces interactions with the real environment, thereby mitigating safety risks.

Our innovative barrier function encodes state-wise safety constraints, ensuring the policy maintains safety at each state. This integration of model-based learning with safety enforcement provides a robust solution for autonomous driving systems. Experimental results in the CARLA simulator demonstrate the effectiveness of our method, outperforming existing approaches in driving score and safety metrics.

The improved generalization capability of our model, evidenced by its performance across various scenarios, highlights the potential for real-world application. Future work will focus on refining the model to handle diverse conditions and bridge the gap between simulation and real-world performance. This study contributes to the progress of safe and reliable autonomous driving systems, aiming for a safer and more efficient transportation future.

## Figures and Tables

**Table 1:** Driving performance and infraction analysis in the testing environment.

Metric	DS (%, ↑)	RC (%, ↑)	IS (↑)	CO (↓)	CP (↓)	CV (↓)	RI (↓)	AB (↓)
Latent SW-PPO	48.22 ± 2.21	58.43 ± 1.32	0.83 ± 0.04	0.08	0.03	0.33	0.09	0.36
SW-PPO	25.98 ± 3.71	44.83 ± 1.43	0.58 ± 0.07	0.62	0.04	0.69	0.72	0.51
Latent PPO	39.71 ± 3.27	55.87 ± 2.26	0.71 ± 0.03	0.43	0.11	0.61	0.79	0.47
Roach PPO	24.54 ± 4.02	48.53 ± 3.24	0.51 ± 0.06	2.47	0.14	2.36	0.54	0.92
PPO	6.721 ± 1.86	14.30 ± 2.87	0.47 ± 0.03	2.37	1.64	1.46	1.59	1.28
SAC	5.084 ± 1.89	12.46 ± 3.78	0.41 ± 0.04	3.35	2.74	1.42	1.89	2.12

We compare our models with several baselines in terms of driving performance and infractions incurred. We report the metrics for 3 evaluation runs of each model on the evaluation setting. For the primary metrics (DS: Driving Score, RC: Route Completion, IS: Infraction Score), we show the mean and std. For the remaining infractions per km metrics (CP: Collisions with Pedestrians, CV: Collisions with Vehicles, CO: Collisions with Others, RI: Red Light infraction, AB: Agent Blocked), we show only the mean. In subsequent experiments, we observed that the std did not exhibit significant variation and did not influence the experimental results. Consequently, std is not reported in the subsequent experiments.

**Table 2:** Driving performance in the training environment.

Town Name	Town03			Town04			Averge (Town03 + Town04)		
Metric	DS (%, ↑)	RC (%, ↑)	IS (↑)	DS (%, ↑)	RC (%, ↑)	IS (↑)	DS (%, ↑)	RC (%, ↑)	IS (↑)
Latent SW-PPO	50.31	64.17	0.78	52.93	65.16	0.81	51.62	64.67	0.80
SW-PPO	32.29	46.71	0.69	34.76	49.21	0.71	33.53	47.96	0.70
Latent PPO	42.46	60.43	0.70	44.99	62.42	0.72	43.73	61.43	0.71
Roach PPO	34.40	59.64	0.58	36.42	60.27	0.60	35.41	59.96	0.59

After the agent is trained on two towns, it is tested on both towns. The average is the average of the driving performance in the two towns.

**Table 3:** Driving performance of different algorithm models on different towns in the testing environment.

Town Name	Town01			Town02			Town05			Town06		
Metric	DS (%, ↑)	RC (%, ↑)	IS (↑)	DS (%, ↑)	RC (%, ↑)	IS (↑)	DS (%, ↑)	RC (%, ↑)	IS (↑)	DS (%, ↑)	RC (%, ↑)	IS (↑)
Latent SW-PPO	48.82	60.23	0.81	52.36	62.34	0.84	44.86	54.08	0.83	46.85	57.08	0.82
SW-PPO	28.26	42.66	0.66	31.26	56.65	0.55	21.63	37.65	0.57	22.43	42.35	0.53
Latent PPO	40.41	58.76	0.69	44.26	60.21	0.74	36.65	51.23	0.72	37.56	53.26	0.71
Roach PPO	25.65	50.46	0.51	27.65	56.32	0.49	20.40	42.23	0.48	24.36	45.12	0.54

## Data Availability

The data in this study are available from the first author upon request.
